# Poly-L-Arginine Induces Apoptosis of NCI-H292 Cells via ERK1/2 Signaling Pathway

**DOI:** 10.1155/2018/3651743

**Published:** 2018-06-14

**Authors:** Ya-Ni Wang, Ling-Ling Zhang, Xiao-Yun Fan, Sha-Sha Wu, Sheng-Quan Zhang

**Affiliations:** ^1^Department of Respiratory and Critical Care Medicine, The Geriatric Institute of Anhui, The First Affiliated Hospital of Anhui Medical University, Number 218 Jixi Road, Hefei, Anhui 230022, China; ^2^Department of Biochemistry and Molecular Biology, Anhui Medical University, Number 81 Meishan Road, Hefei, Anhui 230022, China

## Abstract

Cationic protein is a cytotoxic protein secreted by eosinophils and takes part in the damage of airway epithelium in asthma. Poly-L-arginine (PLA), a synthetic cationic protein, is widely used to mimic the biological function of the natural cationic protein in vitro. Previous studies demonstrated the damage of the airway epithelial cells by cationic protein, but the molecular mechanism is unclear. The purpose of this study aimed at exploring whether PLA could induce apoptosis of human airway epithelial cells (NCI-H292) and the underlying mechanism. *Methods*. The morphology of apoptotic cells was observed by transmission electron microscopy. The rate of apoptosis was analyzed by flow cytometry (FCM). The expressions of the phosphorylation of extracellular signal-regulated kinase 1/2 (ERK1/2), Bcl-2/Bax, and cleaved caspase-3 were assessed by western blot. *Results*. PLA can induce apoptosis in NCI-H292 cells in a concentration-dependent manner. Moreover, the phosphorylation of the ERK1/2 and the unbalance of Bcl2/Bax, as well as the activation of caspase-3, were involved in the PLA-induced apoptosis. *Conclusions*. PLA can induce the apoptosis in NCI-H292 cells, and this process at least involved the ERK1/2 and mitochondrial pathway. The results could have some indications in revealing the apoptotic damage of the airway epithelial cells. Besides, inhibition of cationic protein-induced apoptotic death in airway epithelial cells could be considered as a potential target of anti-injury or antiremodeling in asthmatics.

## 1. Introduction

Bronchial asthma is the 14th important chronic disease that affects approximately 3 million patients among the world. Although the mortality of asthma is declining with effective management, patients without effective control still pose a serious financial and social burden on the health care [1]. Many inflammatory cells and cytokines participate in the process of asthma, and eosinophils can be one of the most important cells. The pathophysiological effect of the eosinophils marked infiltration into the airway and the release of toxic protein especially the cationic protein major basic protein (MBP) which can lead to the damage of the bronchial epithelial cells [2, 3].

The quantity and activity of airway epithelial cells are closely related to the severity of asthma especially to airway hyperresponsiveness and airway remodeling. Firstly, airway epithelium forms the first continuous protective barrier against natural pathogenic microorganisms; the damage of the airway epithelium can increase the susceptibility of the mucosal intrinsic and immune cells to antigen which eventually leads to severe airway hyperresponsiveness (AHR) [4]. Besides, in the pathophysiological process of asthma, the activation of proinflammatory cytokines and the damage of the cells let epithelium in a constant state of damage and repair. This could eventually result in a vicious cycle and occurrence of airway remodeling [5]. There has been much circumstantial evidence implicating that accidents of apoptosis in airway epithelial cells could be an important mechanism which leads to the injury of the epithelial barrier and airway remodeling in asthma, but little is known about the apoptotic damage to the airway epithelium by cationic protein [6–8].

Poly-L-arginine (PLA) is a synthetic cationic polypeptide characterized by its cationic charge with the similar biology and chemical function to MBP and was widely used to mimic the function of MBP in vitro. In the process of eosinophil asthma, with infiltration of eosinophils, cytotoxic cationic proteins were released which finally leads to exfoliation and death of epithelial cells. PLA could stimulate exfoliation and apoptotic death of epithelial cells [9, 10]. However, the underlying molecular mechanism of these cytotoxic cationic proteins to the damage of the airway epithelium is still unclear. In this study, we explored the molecular mechanism of PLA to the damage of the epithelial cells.

## 2. Materials and Methods

### 2.1. Materials

PLA was bought from Sigma-Aldrich (St. Louis, MO, USA). And PD98059, an inhibitor of ERK1/2, was also taken from Sigma-Aldrich. PD98059 was dissolved in dimethyl sulfoxide which was purchased from Sigma-Aldrich. Fetal bovine serum (FBS) was from Gibco (Australian origin). Anti-ERK1/2 and anti-phospho-ERK1/2 were from ImmunoWay Biotechnology (Plano, TX, USA). Anti-Bax, anti-Bcl-2, and anti-caspase-3 were from Cell Signaling Technology (Danvers, MA, USA). Anti-*β*-actin was acquired from Zhongshanjinqiao Biotechnology (Beijing, China). Annexin V-FITC Apoptosis Detection Kit with propidium iodide (PI) double staining flow cytometry was purchased from Bestbio Biotechnology Company (Shanghai, China).

### 2.2. Cell Culture

NCI-H292 is a kind of human lung mucoepidermoid carcinoma cell line with alveolar type II epithelial cell characteristics. It has monolayer adherent growth status and was from the Chinese Academy of Sciences, Shanghai Institute of Life Sciences Cell Bank. NCI-H292 cells were cultured in RPMI 1640 complete medium (Thermo Fisher Scientific, America) with 10% fetal bovine serum (Life Technologies, Carlsbad, CA, USA) and 100 *μ*g/ml penicillin and 100 *μ*g/ml streptomycin in a humidified 5% CO_2_ atmosphere at 37°C. For experiments, when cells adhere to about 80%, the cells were passaged, digested with trypsin, centrifuged, added 1 ml of culture medium to make cell suspension, then counted under a light microscope by using a cell counter plate (Thermo Fisher Scientific, America), and seeded into six-well plates about 5 × 10^5^ cells per well for cultivation. When the cells in the well converge to 80%, they were treated with PLA at a concentration of 0, 20, 40, or 60 mg/l for 24 h, respectively. In other groups, with previous experiments, the cells were pretreated with the inhibitor of ERK1/2 PD98059 for 30 min.

### 2.3. Transmission Electron Microscopy

The NCI-H292 cells were treated with 0 or 60 mg/l of PLA for 24 h. Then, the cells were digested with trypsin and centrifuged at 1000 rpm for 3 min. The supernatants were collected. The cell pellets were fixed in 2.5% glutaraldehyde for 5 h. After removing the fixative, each cell mass was washed twice by cold phosphate-buffered saline (PBS), then dehydrated with graded ethanol. After dehydration, each cell mass was immersed with propylene oxide and finally was embedded in epoxy resin. After incubation in citrate for 15 min, thin sections of 50–70 nm thickness were prepared, and the sections were observed directly under transmission electron microscopy (TEM; JEM-1230, Japan).

### 2.4. Flow Cytometric Analysis

The NCI-H292 cells were digested and made into a suspension, then counted by a cell counter plate under the microscope (TS100, Nikon, Japan) and seeded into a six-well culture plate for 15 × 10^4^ cells per well. The cells were exposed to increasing concentrations of PLA (0, 20, 40, and 60 mg/l), respectively, for 24 h. In another group, the cells were grouped into control, PD98059 (PD), PLA, and PLA + PD groups. With the experiments, the cells in groups PD and PLA + PD were pretreated with PD98059 20 *μ*M for 30 min. After pretreatment with PD98059, 20 mg/l PLA was added into the group of PLA and PLA + PD for 24 h, then the cells were collected into centrifuge tubes, washed with 400 *μ*l annexin V, and suspended for 10–15 min. After staining with 10 *μ*l of PI staining solution, the cells were incubated for 5 min in the laser eight-color flow cytometer, and each tube was shaken evenly. The fluorescence of FITC was detected by a wavelength of 515 nm passband filter, and PI was detected by the other filter with a wavelength over 560 nm. Then, the parameters were set and the analysis area was selected as following: the abscissa was set as FITC to represent annexin V-FITC and the vertical coordinate was set as PE-Texas which represents the PI. The cells and apoptotic cells were evaluated by a quadrant gate tool.

For the result determination, the apoptosis rate was analyzed by Flow Jo software (BD FACSVerse, the USA). On the flow cytometer bivariate scatter plot, the normal living cells were on the lower left quadrant which was FITC−/PI−, and the necrotic cells were on the upper left quadrant of which was FITC−/PI+; the lower right quadrant represented the early apoptotic cell which showed FITC+/PI−, and the upper right quadrant was the late apoptotic cells which showed FITC+/PI+.

### 2.5. Western Blot

The NCI-H292 cells were divided into five groups with concentrations of 0 (control), 10, 20, 40, or 60 mg/l PLA, respectively. Others were divided into the control group, PD group, PLA group, and PLA + PD group. The whole-cell proteins were extracted with RIPA buffer (0.1% SDS, 1% Nonidet P-40, 150 mM NaCl, 0.5% deoxycholic acid, and 50 mM Tris-HCl, pH 7.4) which contained the protease inhibitor phenylmethanesulfonyl fluoride. 12% sodium dodecyl sulfate polyacrylamide gel electrophoresis (SDS-PAGE) was used to resolve the total cellular protein, and then the protein was transferred to polyvinylidene fluoride (PVDF) membranes. Next, 5% nonfat milk 2.5 g was used and dissolved in 50 ml Tris-buffered saline with Tween 20 (TBS-T) to block the membranes at room temperature for 2 h. After washing the membranes with TBS-T three times and 10 min per wash, the membranes were incubated with rabbit anti-human antibodies against ERK1/2, p-ERK1/2, Bcl-2, Bax, and caspase-3 or mouse anti-human *β*-actin antibody (all at 1 : 500 dilutions), respectively, at 4°C overnight. In the next morning, the membranes were as mentioned before and incubated with the appropriate horseradish peroxidase-conjugated goat anti-rabbit or goat anti-mouse secondary antibodies at a certain degree of dilution (1 : 5000 for ERK1/2, Bax, and caspase-3; 1 : 8000 for p-ERK1/2 and Bcl-2; and 1 : 10000 for *β*-actin) for 2 h at room temperature. Finally, they were washed with TBS-T, and protein immunostaining was observed by enhanced chemiluminescence (ECL, Thermo Scientific).

### 2.6. Statistical Processing

All experiments were repeated three times. SPSS version 16.0 (IBM, Armonk, NY, USA) was used to analyze statistics. All values were tested for normality and shown as mean ± standard. For more than two sets of comparisons, one-way analysis of variance (ANOVA) was applied. When assuming that the variance between the two groups is equal, Fisher's least significant difference test (LSD-t) was used, while for not equal, Dunnett's T3 was used. It is statistically different when *P* values are less than 0.05.

## 3. Results

### 3.1. PLA Altered the Morphological Characteristics of NCI-H292 Cells

**Figure 1:**
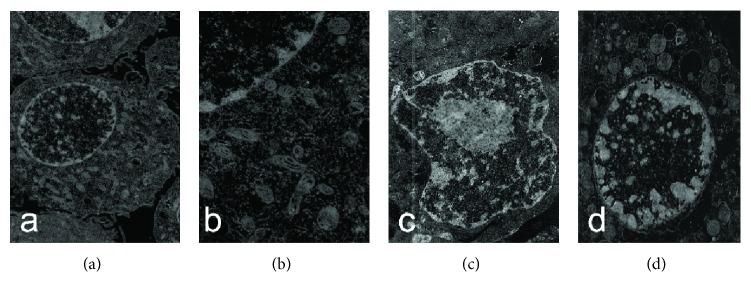
Effects of PLA on the morphology of NCI-H292 cells. (a) Control cells were flattened; the cytoplasm stretched to the periphery and adhered to the growth of the support (×8000). (b) In the control group, the mitochondrial ridge displayed continuous integrity and was regularly arranged (×20000). (c) After treatment by PLA, the surface of the nuclear membrane was uneven and the chromosomes were concentrated under it (×8000). (d) After treatment by PLA, the mitochondria were round and empty (×20000).

The morphology of the cells was observed under transmission electron microscopy. In the control group, the morphology of NCI-H292 cells was typical for epithelial cells. The nuclear membrane was complete and the border was clearly visible (Figure 1(a)). The mitochondrial morphology was normal and cristae were arranged regularly (Figure 1(b)). In contrast, cells exposed to 60 mg/l of PLA for 24 h showed typical apoptotic changes in morphology. The nucleus membrane shrank and the chromatin was concentrated under the nuclear membrane (Figure 1(c)). Besides, mitochondria swelled and cristae became disordered and irregular (Figure 1(d)).

### 3.2. PLA Induces Apoptosis in NCI-H292 Cells in a Concentration-Dependent Manner

**Figure 2:**
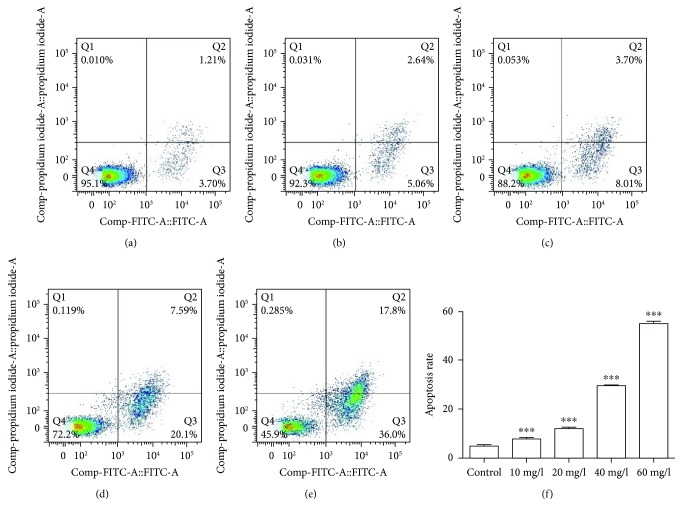
Apoptosis rates in NCI-H292 cells after PLA exposure for 24 h. (a) Control group, (b) 10 mg/l PLA group, (c) 20 mg/l PLA group, (d) 40 mg/l PLA group, and (e) 60 mg/l PLA; (f) the apoptotic rate bar graph. With the increase concentration of PLA, the apoptosis rate of NCI-H292 cells increased. Compared with the control group, ^∗∗∗^*P* < 0.001.

Annexin V-FITC/PI double staining assays were used to evaluate the apoptosis induced by PLA following the manufacturer's instructions, which was then measured by flow cytometer. PLA significantly promoted apoptosis of NCI-H292 cells. The apoptosis rates in NCI-H292 cells have increased after treatment of PLA (Figures 2(a)–2(e)). The proportion of annexin V-positive/PI-negative cells increased from 7.82% to 55.23% after being treated with increased doses of PLA for 24 h. Compared with 4.97% in the control group, the differences at each concentration of PLA were statistically significant (*P* < 0.001; Figure 2(f)). In conclusion, the consequences were in agreement with earlier results that the apoptosis happened to cells by PLA. Furthermore, the induction of apoptosis in NCI-H292 by PLA was in a concentration-dependent manner.

### 3.3. The Effect of PLA to the ERK1/2 Pathway in NCI-H292 Cells

**Figure 3:**
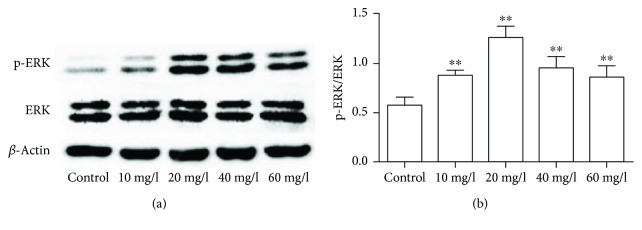
Expression of p-ERK/ERK in NCI-H292 cells. (a) Protein expression bands were detected by western blot. (b) The bar chart shows the ratio of p-ERK/ERK. Compared with the control group, phosphorylation of ERK1/2 increased significantly in groups treated with PLA, especially at a PLA concentration of 20 mg/l. ^∗∗^*P* < 0.01.

There is much evidence to show that mitogen-activated protein kinases (MAPK) play an important role in cell proliferation, differentiation, and apoptosis [11]. Extracellular signal-regulated kinase (ERK1/2) signaling pathway was the earliest discovered classical signal transduction pathway of Ras-Raf-MAPK involved in the process of cell apoptosis [12]. Our results showed that ERK1/2 can be phosphorylated by PLA. When the concentration of PLA was 10 mg/l, p-ERK/ERK achieved statistical significance. While at a PLA concentration of 20 mg/l, p-ERK/ERK has reached its peak value. (*P* < 0.01; Figures 3(a) and 3(b)).

### 3.4. The Effects of PLA to the Expressions of Antiapoptosis and Proapoptosis Proteins in NCI-H292 Cells

**Figure 4:**
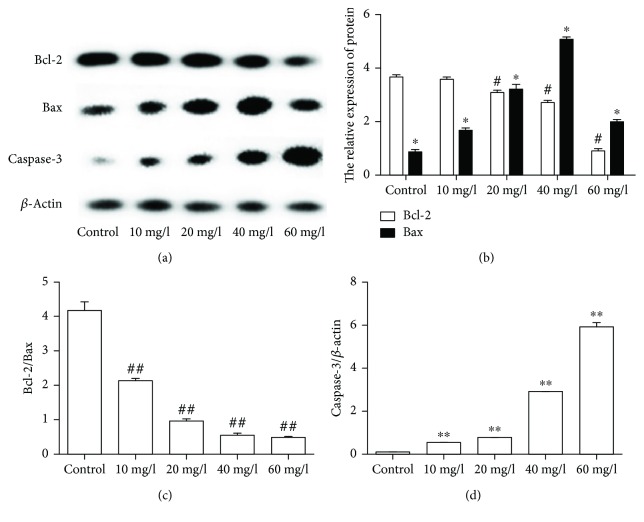
Expression of Bax, Bcl-2, and caspase-3 in NCI-H292 cells. (a) Protein expression bands were detected by western blot. (b) The bar charts of Bax/*β*-actin and Bcl-2/*β*-actin. (c) The bar chart shows the ratio of Bcl-2/Bax. (d) Expression of caspase-3 normalized to *β*-actin. Compared with the control group, the expression of Bcl-2/Bax decreased and cleaved caspase-3 increased. ^#^*P* < 0.01, ^##^*P* < 0.01, ^∗^*P* < 0.01, and ^∗∗^*P* < 0.001.

The molecular mechanism of apoptosis is complicated. The Bcl-2 family includes antiapoptosis protein like Bcl-2 and proapoptosis protein like Bax. The imbalance between the Bcl-2 families can stimulate the release of cytochrome c and ultimately activates caspases such as caspase-3 which eventually promotes apoptotic death of cells [13]. As our results showed (Figure 4), at a PLA concentration of 20 mg/l, the decrease of Bcl-2 presented a statistical difference. At a PLA concentration of 10 mg/l, the increase of Bax and the decrease of Bcl-2/Bax presented a statistical difference. Besides, the expression of caspase-3 increased with an increased concentration of PLA. In conclusion, the balance between proapoptosis protein and antiapoptosis protein was destroyed by PLA in NCI-H292 cells, and this further activated caspase-3.

### 3.5. PD98059 Inhibits PLA-Induced ERK1/2 Phosphorylation and Apoptosis in NCI-H292 Cells

**Figure 5:**
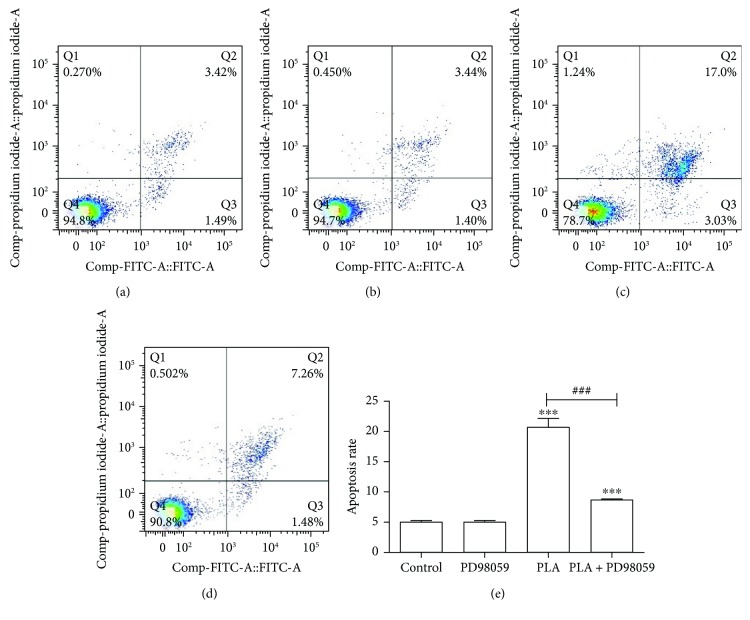
Apoptosis of NCI-H292 cells treated with PLA and PD98059. (a) Control group, (b) PD group, (c) PLA group, and (d) PLA + PD group. (e) The apoptotic rates of the control group, PD group, PLA group, and PLA + PD group. Compared with the control group, apoptosis in NCI-H292 cells was increased by PLA; ^∗∗∗^*P* < 0.001. Compared with the PLA group, the apoptosis rate decreased in the PLA + PD group; ^###^*P* < 0.001.

In order to explore the association between ERK1/2 and apoptosis in PLA-treated cells, we pretreated the cells with PD98059 (20 *μ*M), the specific inhibitor of ERK1/2 [11]. The apoptosis rate of NCI-H292 cells in the PD98059 (20 *μ*M) group was 8.67%, which was significantly lower than the 20.72% apoptosis rate in the PLA group. Besides, PD98059 can inhibit PLA-induced apoptosis in NCI-H292 cells, and the apoptosis rate of NCI-H292 cells decreased and presented statistically significant in the PLA + PD group in contrast to the PLA group (*P* < 0.001; Figure 5).

### 3.6. The Effects of Inhibition of ERK1/2 to the Expressions of Apoptosis-Related Proteins

**Figure 6:**
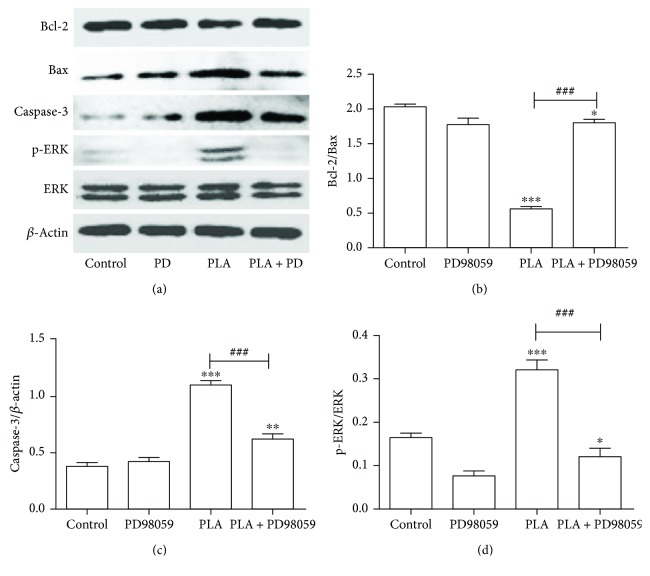
Expression of Bax, Bcl-2, caspase-3, and p-ERK/ERK in NCI-H292 cells treated with PLA and PD98059. (a) Protein expression bands were detected by Western blot. (b) The bar chart of Bcl-2/Bax. (c) The bar chart of caspase-3/*β*-actin. (d) The bar chart of p-ERK/ERK. Compared with PLA group, PD98059 significantly inhibited the activation of ERK1/2 and also blocked the regulatory effect of PLA on Bcl-2/Bax and cleaved caspase-3. ^###^*P* < 0.001. Compared with the control group, ^∗^*P* < 0.05, ^∗∗^*P* < 0.01, ^∗∗∗^*P* < 0.001.

Since apoptotic cells decreased in the group pretreated with PD98059, we further explored the mechanism of such effects. In contrast with the PLA group, as the phosphorylation of ERK1/2 was inhibited by PD98059, the expression of proapoptotic protein Bax was decreased and the expression of antiapoptotic protein Bcl-2 was restored (Figure 6(a)). And the ratio of Bcl-2/Bax was similar to the blank control group (Figure 6(b)). Besides, the activation of downstream apoptotic molecules caspase-3 was reduced (*P* < 0.001; Figure 6(c)). That is to say, the inhibition of ERK1/2 can decrease PLA-induced apoptosis in NCI-H292 cells.

## 4. Discussion

The airway epithelial cell as a protective barrier plays an important role in the respiratory system. And the damage of the airway epithelium has been considered as an important etiology of asthma [14]. Major basic protein (MBP) derived from the eosinophils is a natural cationic polypeptide mainly containing arginine and lysine. MBP is considered as one of the markers in eosinophil asthma, and the secretion of MBP contributes to the damage of epithelial cells. Poly-L-arginine (PLA) is widely used to study the function of MBP in vitro. It has already been reported that PLA can induce apoptosis-like changes in airway epithelial cells. PLA can blur the nucleus membrane, resulting in shrinkage and condensation of the nucleus. In our study, we firstly determined that PLA contributes to apoptosis morphology changes in NCI-H292, and this was in consistence with the previous studies [9, 15]. But the potential molecular mechanism seems unclear and few studies have reported it. Hence, we further explored the signal molecules involved in this process.

Mitogen-activated protein kinases (MAPK) are an important signaling pathway in many cell functions. There are more than 20 subtypes of MAPK in mammals and ERK1/2 is one of the most widely studied members. Much evidence has showed the high activation of ERK1/2 in asthma, and the phosphorylation of ERK1/2 has played a role in the activation of apoptosis [16, 17]. It has been reported that the phosphorylation of p38 MAPK is related to the inhibited growth of breast cancer [18].

That is to say, different members of the MAPK family play different roles in cells. And in this study, we found that PLA-induced apoptosis in NCI-H2929 cells was accompanied by the activation of ERK1/2.

Apoptosis acts a pivotal part in maintaining homeostasis between cell survival and death. The permeability of mitochondrial membrane has been shown to play a role in apoptosis. Increased mitochondrial membrane permeability eventually leads to the release of cytochrome c and activation of caspase cascades. The Bcl-2 family involves antiapoptotic members like Bcl-2 and Bcl-Xs and proapoptotic members like Bax, Bad, Bak, and Bcl-Xs. They have played a role in sustaining the stability of mitochondrial membrane and the release of cytochrome c [18, 19]. Caspase-3 is called “death-executing protease” which is located in the downstream of Bcl-2 family and occupies a central position of apoptosis [20]. Bax can promote the release of cytochrome c from the mitochondria to the cytosol which eventually contributes to the activation of caspase-3. When Bax is overexpressed in cells, the number of Bax/Bax homodimers increases and cells become more allergic to death signals. While the expression of Bcl-2 increases, the Bax/Bax dimers dissociate in large amounts and produce a more stable Bcl-2/Bax heterodimer to fight against apoptosis so as to prolong cell survival [21, 22]. The imbalance of Bcl-2/Bax leads to many dysfunctions such as the increase of mitochondrial membrane permeability and intracellular calcium as well as oxidative stress. All these can affect the cell's respiratory function and induce cell apoptosis [23]. Therefore, the balance between these two functionally opposite proteins is the key to cell survival [24]. In this study, the decrease of Bcl-2 and the increase of Bax were discovered in groups with PLA especially at a PLA concentration of 40 mg/l. Moreover, the rate of Bcl-2/Bax decreased in a dose-dependent manner. Hence, we postulate that PLA can destroy the balance between Bcl-2 and Bax and further activate caspase-3 cascades. Then, we measured the expression of caspase-3. The results were in consistence with our expectations. The expression of caspase-3 increased in a concentration-dependent manner in groups that were treated with PLA. In order to further verify the apoptosis in NCI-H292 cells induced by PLA, in vitro, we quantitatively analyzed the apoptosis rate by FCM. As the results showed, apoptosis in NCI-H292 cells induced by PLA was in a dose-dependent manner. All these at least could demonstrate that PLA-induced apoptosis in NCI-H292 cells in a concentration-dependent manner, and this process involved the activation of the mitochondrial pathway.

The ERK pathway has been shown to directly affect mitochondrial function by inhibiting mitochondrial respiration [25] and reducing mitochondrial membrane potential [26]. The phosphorylation of ERK1/2 leads to the disruption of mitochondrial membranes and release of cytochrome c [27, 28]. The activation of ERK1/2 can upregulate a proapoptotic protein, such as Bax [29, 30], and downregulate an antiapoptosis protein such as Bcl-2 [31, 32], which facilitate the release of cytochrome c and apoptosis. But whether the increase of Bax and decrease of Bcl-2 protein in the PLA-treated NCI-H292 cells were through ERK1/2 signal remains unclear, so we inhibited the ERK1/2 by PD98059. In our study, we found that PLA-induced apoptosis in NCI-H2929 cells was accompanied by the activation of ERK1/2. And in order to explore the underlying association between ERK1/2 and apoptosis, we use the inhibitor of ERK1/2 PD98059 to explore the association between ERK1/2 and apoptosis-related protein. As the results showed, the inhibition of ERK1/2 can restore the antiapoptotic protein Bcl-2 and downregulated the proapoptotic protein Bax. All these eventually lead to a reduced expression of the apoptosis protein caspase-3 and decreased apoptosis rate. Hence, we concluded that PLA can activate ERK1/2 to promote apoptosis in NCI-H292 cells, and the inhibition of ERK1/2 by PD98059 can suppress the apoptotic damage in cells, and this could provide a new therapeutic target of epithelial damage in asthmatics.

In summary, PLA can induce apoptosis in NCI-H292 cells in a concentration-dependent manner. And through our study, we could at least conclude that this process involves the activation of ERK1/2 signaling pathway and mitochondrial pathway. The phosphorylation of ERK1/2 leads to the imbalance of the proapoptotic protein like Bax and antiapoptotic protein like Bcl-2. Hence, the ratio of Bcl-2/Bax altered in favor of apoptosis. And this further facilitates the expression of apoptosis protein caspase-3. That is to say, PLA can activate mitochondrial-related apoptosis signaling pathway through ERK1/2 in NCI-H292 cells. In addition, the inhibition of ERK1/2 could reverse the unbalance between the antiapoptotic protein and the proapoptotic protein. However, it is unclear whether other signaling pathways have been involved in PLA-induced apoptosis. Future research could be explored for the mechanism of airway epithelial cell injury induced by PLA in a broader context, which can provide additional solutions for the remission and treatment of eosinophil asthma.

## Data Availability

The data used to support the findings of this study are available from the corresponding author upon request.
